# Burden of Suicidal Ideation and Attempt among Persons Living with HIV and AIDS in Semiurban Uganda

**DOI:** 10.1155/2016/3015468

**Published:** 2016-03-17

**Authors:** Godfrey Zari Rukundo, Brian Leslie Mishara, Eugene Kinyanda

**Affiliations:** ^1^Department of Psychiatry, Mbarara University of Science and Technology, Mbarara, Uganda; ^2^Centre for Research and Intervention on Suicide and Euthanasia and Psychology Department, Université du Québec à Montréal, Montréal, QC, Canada H3C 3P8; ^3^Uganda Medical Research Council, Entebbe and Department of Psychiatry, Makerere College of Health Sciences, Kampala, Uganda

## Abstract

Although the impact of HIV/AIDS has changed globally, it still causes considerable morbidity and mortality, including suicidality, in countries like Uganda. This paper describes the burden and risk factors for suicidal ideation and attempt among 543 HIV-positive attending two HIV specialized clinics in Mbarara municipality, Uganda. The rate of suicidal ideation was 8.8% (*n* = 48; 95% CI: 6.70–11.50) and suicidal attempt was 3.1% (17, 95% CI 2.00–5.00). The factors associated with increased risk for suicidal ideation and attempts were state anger (OR = 1.06, 95% CI: 1.03–1.09; *p* = 0.001); trait anger (OR 1.10, 95% CI 1.04–1.16, *p* = 0.002); depression (OR 1.13, 95% CI 1.07–1.20, *p* = 0.001); hopelessness (OR 1.12, 95% CI 1.02–1.23, *p* = 0.024); anxiety (OR 1.06, 95% CI 1.03–1.09); low social support (OR 0.19, 95% CI 0.07–0.47, *p* = 0.001); inability to provide for others (OR 0.19, 95% CI 0.07–0.47, *p* = 0.001); and stigma (OR 2.48, 95% CI 1.11–5.54, *p* = 0.027). At multivariate analysis, only state anger remained statistically significant. HIV/AIDS is associated with several clinical, psychological, and social factors which increase vulnerability to suicidal ideation and attempts. Making suicide risk assessment and management an integral part of HIV care is warranted.

## 1. Introduction

Suicide remains an underreported phenomenon in the developing world despite its possible high prevalence [1]. Studies have reported different suicide prevalence rates in several countries and settings [2–4]. Physical illness, mental disorders, and social-environmental factors are associated with increased suicide risk [5]. The relationship between HIV/AIDS and suicidal ideation and attempts has been discussed for almost three decades but findings have been inconsistent [6]. Whereas some studies have reported high rates of suicide among persons with HIV/AIDS, other studies have reported no significant increased suicide risk [7–12]. Furthermore, there is paucity of information about this topic in Sub-Saharan Africa [13] despite the fact that Sub-Saharan Africa accounts for more than two-thirds of the HIV global disease burden [14, 15]. There is need for a local body of research to inform policy development and programme implementation concerning suicidal ideation and attempts in persons with HIV/AIDS in Sub-Saharan Africa [16]. This study investigated the burden of suicide and risk factors associated with suicidal ideation and attempts among African individuals living with HIV/AIDS in semiurban Uganda.

## 2. Materials and Methods

We conducted a cross-sectional survey at the Immune Suppression Syndrome Clinic of Mbarara Regional Referral Hospital (MRRH) and The AIDS Support Organization (TASO) Mbarara branch clinic. Both clinics are located in Mbarara municipality, South Western Uganda, and provide care to HIV-positive patients. The study participants were 15 years or older, HIV seropositive, receiving care at either the TASO or ISS clinic, and each participant provided informed consent. The exclusion criteria consisted of attending the clinic for the first time and not yet being enrolled for care, being physically very ill, and refusal to participate in the study. The participants were not given any monetary incentive.

Of the 573 clients who were contacted to participate in the study, 5% (*n* = 30) of the patients refused to participate. The most common reasons for nonparticipation was coming from too far, their employers having given limited time for the clinic visit, and associating participation in the study with the stigma of mental illness.

Since some of the participants could not read and write, the questionnaires were interviewer administered for consistency. All questionnaires were translated into the locally spoken language (Runyankore-Rukiga) by an expert translator, and they were later back translated by another expert in translation. The quality of the translation and back translation was checked by two people, a psychiatric nurse and a social worker, who both spoke both English and Runyankore fluently.

Screening for suicidal ideation and attempts was conducted using five questions about death, suicidal ideation, and attempts, four of which were used in the study on Suicide Attempts in the Epidemiologic Catchment Area Study by Moscicki and colleagues in the United States [17]: (1) Have you thought a lot about death in the past? (2) Have you felt like you wanted to die in the past? (3) Have you felt so low and thought about committing suicide in the past year? (4) Did you attempt suicide in the past year? (5) Have you ever attempted suicide at some other time in life? A participant who answered yes to any of the questions (3)–(5) was considered to have suicidal ideation and an attempt. Because of the state of illness of the participants, in order to minimize respondent fatigue due to lengthy interviews, we randomly assigned some of the respondents to either the psychological or the social assessment modules of this study.

### 2.1. Study Instruments

The WHO clinical staging and CD4 counts were used to determine the stage of HIV infection at the time of the study, based upon the patients' clinical records, classifying patients according to four stages of infection, I, II, III, and IV.

The Beck Hopelessness Scale [18] was used to assess the extent of hopelessness in the participants who answered the questionnaire on psychological factors (substudy I). This 20-item questionnaire assesses negative expectations and pessimism about one's future. Each statement is scored one point. The total of responses to the true-false questions result in scores which are rated follows: 0–3 minimal hopelessness, 4–8 mild, 9–14 moderate, and 15–20 severe hopelessness.

The CAGE questionnaire [19], consisting of four “yes-no” questions, was used to screen for alcohol abuse and dependence. It was administered to all participants in the study. The person is asked if he/she has considered cutting down the amount of alcohol consumed, if she/he gets annoyed when people comment on the drinking habit, if the person feels guilty about the amount drunk and the habit, and if she/he takes an early morning alcoholic drink as an eye opener. In the current study, we used a cut-off of two out of the four questions to indicate alcohol abuse and dependence.

The relationship between suicidality and the way participants solved their problems was assessed with the 20-item Problem Solving Styles Questionnaire [20] that describes styles of reacting to problems to reduce, remove, or tolerate stress. The items in the questionnaire have been categorized according to four main ways in which people respond to problems: sensing, intuitiveness, feeling, and thinking. It is believed that in solving problems, it matters first how we perceive information. This is usually through the process of sensing (through the five senses) and through intuition (the ability to know things without the use of rational thinking processes). This questionnaire uses a five-point Likert scale where 1 = strongly disagree; 2 = slightly disagree; 3 = not sure; 4 = slightly agree; and 5 = strongly agree. The lowest score is 20 and the highest score is 100. The higher the score on each subscale is, the more likely the person uses that way of coping. The Cronbach alpha for this questionnaire in the current study was 0.900.

The 66-item Ways of Coping Questionnaire [21] was administered to assess the ways of participants cope with problems. Each item is scored on a four-point scale: 0, does not apply or not used; 1, used somewhat; 2, used quite a bit; 3, used a great deal. Fifty items of the questionnaire have been used and found to consistently identify eight subscales: confrontive coping, distancing, self-controlling, seeking social support, accepting responsibility, escape avoidance, planful problem solving, and positive reappraisal. The lowest overall score is zero while the highest is 198, with higher scores indicating more use of that way of coping. The Cronbach alpha for this tool in the current study was 0.974.

The Beck depression inventory [22] was used to assess depression symptoms. The final assessment was undertaken by the first author, who is a psychiatrist. Each item is rated on a four-point scale (0 to 3) and total scores are between 0 and 63. The total scores measure the severity of self-reported depression with 0–9 minimal, 10–16 mild, 17–29 moderate, and 30–63 severe.

The Beck Anxiety Inventory [23] was used to assess anxiety symptoms. This 21-item self-report inventory has items rated on a four-point scale (0 to 3), with the total score being between 0 and 63, indicating the severity of anxiety: 0–7 minimal anxiety, 8–15 mild, 16–25 moderate, and 26–63 severe anxiety.

Self-esteem was assessed using the 10-item Rosenberg's Self-Esteem Scale [24]. Each of the 10 items is scored on a 4-point scale: 1 = strongly agree; 2 = agree; 3 = disagree; 4 = strongly disagree. The self-esteem score is calculated after reversing the positively worded items with higher scores indicative of higher self-esteem. The lowest score is 10 and the highest score is 40, a score below 20 indicating low self-esteem. Cronbach's alpha for this instrument was 0.781.

The 20-item State-Trait Anger Scale developed by Spielberger [25] was administered to assess the predisposition to experience angry feelings as a personality trait (trait anger) and the intensity of anger as an emotional state (state anger) at the time of assessment. It assesses the intensity of anger at a particular moment and the frequency of anger experience, expression, and control [25]. Trait anger is scored on a four-point scale with 1 being the least (almost never) and 4 being the most common feeling. The state anger scale is also measured on a four-point scale with 1 (not at all) being the least intense and 4 being the most intense (very much). This questionnaire was administered to participants in substudy I. The questionnaire had a Cronbach alpha of 0.894 and 0.917 for trait and state anger, respectively.

The 16-item Perceived Social Support Questionnaire (PSSQ) was designed by the first author using items outlined in the questionnaire developed by Sarason and colleagues [26, 27]. It assesses four aspects of social support: whether the participant needed support from family and friends, received support, was needed for support by other people, and felt they were able to provide support to others. The social support considered was both practical (physical/material/financial) and moral support. The questionnaire had a Cronbach alpha of 0.970.

The HIV Stigma Scale Questionnaire (HSSQ), a 37-item questionnaire, was designed by the first author using items outlined by Berger and collegues [28]. Thirty-five of the items required answers of “yes” and “no” whereas two of them required explanation. The questionnaire examines, from the patient's perspective, feelings (felt stigma) about being HIV-positive. Questions focus on HIV testing as well as on the feelings and attitudes about HIV-positive status. The questionnaire had a Cronbach alpha of 0.788.

Participants' medical records, carried by the patients, were reviewed to note if there was any additional important information, especially the duration of the HIV diagnosis, physical diagnosis, medications, CD4 cell counts, and the clinical staging of the HIV infection. This was done with permission and consent from the individual participants.

Data analysis was guided by the modified stress-diathesis model in which HIV/AIDS is considered to cause considerable stress that acts on the various vulnerability factors to cause several complications/effects [29]. The stress-diathesis model suggests that an individual's biological vulnerabilities to certain mental disorders can be triggered by stress. This means that if an individual has a high level of vulnerability, it would take lower levels of stress for symptoms of the disorder to develop. In our study population, HIV was a key vulnerability factor whereas suicidal ideation/attempts were the key complications/effects. Results were considered statistically significant if the *p* value was ≤ 0.05 at 95% confidence interval. Cross tabulations and logistic regression analysis were used to assess the relationship between each of the correlates with suicidal ideation and attempts.

The study was approved by the Institutional Research and Ethics Committee (MUST-REC) of Mbarara University of Science and Technology and the leadership at the two study centres. Written informed consent was obtained from each adult participant. Participants below 18 years were contacted to give assent and then the primary caregivers provided written informed consent.

All participants found to have moderate-severe psychiatric illness or increased risk of suicidality were referred to the mental health unit of Mbarara hospital for appropriate treatment. The psychiatric illness and risk for suicidality were determined by high scores on the screening instruments. The referral was done in collaboration with the case managers at the two clinics. The total number of participants that required referral for further assessment and treatment was eight.

## 3. Results

A total of 543 HIV-positive individuals were interviewed. Forty-four percent (*n* = 239) of the participants attended the TASO clinic while 56% (*n* = 304) attended the ISS clinic. Among the participants, 24% (*n* = 131) were males and 76% (*n* = 412) were females. The participant ages ranged from 15 to 70 years with a median of 36.0 years and a mean age of 36.7 years (SD = 9.7). The mean age of the males was significantly higher than that of the females (mean age of males = 39.4 years, SD = 10.6; mean age of females = 35.8 years, SD = 9.1; *t*-test = 3.462, and *p* = 0.001).

More females (76%) than males (60%) had no formal education or only a primary level education (OR 1.65; 95% CI: 1.26–2.15; *p* = 0.001). There was a statistically significant difference between males and females on employment status (OR = 1.81, 95% CI 1.22–2.69 and *p* = 0.003), with the majority (60%) of the females being in the categories of peasants or the unemployed.

### 3.1. Prevalence of Suicide Ideation and Suicide Attempt in HIV/AIDS

Suicidal ideation in the preceding year was reported by 8.8% (*n* = 48; 95% CI: 6.70–11.50) while attempted suicide in the same period was 3.1% (17, 95% CI 2.00–5.00). In addition, 3% (*n* = 16) had attempted suicide at some other time in their life. In total, 10% of the study participants (*n* = 54; 95% CI: 5.00–15.00) met criteria for suicidality defined as suicidal ideation in the preceding year or attempted suicide in one's life time.

### 3.2. Psychological Factors Associated with Suicidal Ideation and Attempts in HIV/AIDS

The psychological factors significantly associated with suicidal ideation and attempts (Table 1) were state anger, trait anger, depression hopelessness, anxiety, and low self-esteem. Only state anger remained significantly associated with suicidal ideation and attempts in this study (Table 1) in the multiple regression analysis.

### 3.3. Problem Solving Styles in HIV/AIDS

As shown in Table 2, the sensing style of information acquisition and analysis was associated with a decrease in the odds for suicidal ideation and attempt (suicidal group mean score = 19.00 (SD = 2.71), nonsuicidal group mean score = 20.62 (SD = 3.33), OR = 0.87, 95% CI 0.77–0.98, and *p* = 0.024). The feeling style was also associated with a decrease in the odds of suicidal ideation and attempt (suicidal group, mean score = 19.68 (SD = 3.34), nonsuicidal group, mean score = 21.16 (SD = 3.15), OR = 0.88, 95% CI 0.78–0.99, and *p* = 0.034).

### 3.4. Ways of Coping in HIV/AIDS

Table 3 shows the coping processes that were assessed. The coping styles found to be adaptive against suicidal ideation and attempts in this study were seeking social support (suicidal group, mean score = 7.14 (SD = 4.84), nonsuicidal group, mean score = 9.66 (SD = 4.60), OR = 0.89, 95% CI 0.83–0.97, and *p* = 0.006), escape avoidance (suicidal group, mean score = 4.17 (SD = 4.42), nonsuicidal group, mean score = 6.53 (SD = 5.41), OR 0.91, 95% CI 0.84–0.99, and *p* = 0.027), planful problem solving (suicidal group, mean score = 4.76 (SD = 3.82), OR 0.89, 95% CI 0.79–0.99, and *p* = 0.036), positive reappraisal (suicidal group, mean score = 5.57 (SD = 4.19), nonsuicidal group, mean score = 8.24 (SD = 5.11), OR 0.90, 95% CI 0.83–0.97, and *p* = 0.009), and distancing (suicidal group, mean score = 3.21 (SD = 3.28), OR 0.89, 95% CI 0.80–0.99, and *p* = 0.026). After adjusting for significant clinical factors, none of the investigated ways of coping style factors remained significantly associated with suicidal ideation and attempts.

### 3.5. Social Factors Associated with Suicidal Ideation and Attempt in HIV/AIDS

#### 3.5.1. Social Support in HIV/AIDS

Participants who reported that they needed support from friends and family had lower odds for suicidal ideation and attempt (OR 0.30, 95% CI 0.10–0.89, and *p* = 0.030) than individuals who felt they did not need support from other people (see Table 3). Similarly, individuals who felt that they were able to provide for their families and friends had lower odds for suicidal ideation and attempt (OR 0.19, 95% CI 0.07–0.47, and *p* = 0.001) than those who felt that they had failed in that responsibility.

#### 3.5.2. Stigma in HIV/AIDS

Among the investigated stigma factors, isolating self from friends and family (OR 0.25, 0.10–0.65, and *p* = 0.004), feeling ashamed of the HIV-positive status (OR 2.48, 95% CI 1.11–5.54, and *p* = 0.027), and being assaulted by a spouse (OR 5.42, 95% CI 1.28–22.96, and *p* = 0.027) were associated with higher odds for suicidal ideation and attempts, as shown in Table 4.

#### 3.5.3. Clinical Factors and Suicidality in HIV/AIDS

Most (76%, *n* = 413) of the participants had experienced excellent or good physical health in the past three months and 8% (*n* = 45) had to cut down on activities because of physical illness. Most of the participants (83%, *n* = 452) were in Stages I and II while 13% (*n* = 70) were in Stage III and 4% (*n* = 21) in Stage IV of the disease. On most recent CD4 cell counts, 16% (*n* = 89) had 0–200 cells/*μ*L, 45% (*n* = 244) had 201–500 cells/*μ*L, and 23% (*n* = 127) had ≥ 501 cells/*μ*L, while 15% (*n* = 83) had never had their CD4 counts done. Seventy percent (*n* = 382) were on ARVs while 30% (*n* = 161) were ARV naïve. The following clinical factors were significantly associated with suicidality: perception of poor physical health (OR 2.22, 95% CI 1.23–3.99, and *p* = 0.007), physical pain (OR 1.83, 95% CI 1.01–3.30, and *p* = 0.049), reducing the work due to illness (OR = 2.22, 95% CI 1.23–3.99, and *p* = 0.004), and duration of HIV infection (those newly infected having higher proportions with suicidality than those who had lived longer with the infection) (OR 1.02, 95% CI 1.01–1.03, and *p* = 0.001). All clinical factors that were significantly associated with suicidality in bivariate analysis were included in the model for the subsequent analyses.

### 3.6. Negative Life Events and Suicidal Ideation and Attempt in HIV/AIDS

Negative life events at different stages in life were assessed and scores were generated for each section of negative life events. Only negative life events experienced later in life in relation to children, for example, problems in bringing up your children, were significantly associated with suicidal ideation and attempts (suicidal group, mean score = 2.28 (SD = 6.35), nonsuicidal group, mean score = 1.09 (SD = 1.16), OR 1.02, 95% CI 0.97–1.33, and *p* = 0.006).

## 4. Discussion

The objective of this study was to determine prevalence of suicidality and to explore associated factors among HIV-positive patients in Mbarara municipality. The prevalence of suicidality was 10%. Perceived poor physical health, physical pain, trait/state anger, anxiety, depression, hopelessness, lack of social support, poor problem solving skills, and ways of coping were risk factors associated with increased suicidality. We also demonstrated that poor problem solving skills and lack of social support seem to be a vulnerability factor rather than a precipitant for suicidal ideation and attempt.

### 4.1. Prevalence of Suicidal Ideation in HIV/AIDS

In the current study, the rate of suicidal ideation and attempts was 10% (54/543) with a rate of suicidal ideation of 8.8% in the preceding year. The rate of suicidal ideation and attempts obtained in this study is similar that of 7.8% reported by Kinyanda and colleagues [30] in Entebbe Uganda in similar settings. However, when the rates of suicidal ideation reported in this study are compared to those reported elsewhere, they are much lower. For instance, the following rates have been reported in the west: 27% in the USA [31, 32]; 21% in Australia [33]; 31% in the UK [34]; and 19% in New York, USA [35]. Differences between the rates obtained in this study with those from the west could be attributed to two factors: the first being methodological differences in the assessment of suicidal ideation and attempts in this study compared to the other studies undertaken in the west. Despite the methodological differences, the large difference between the rates reported in Uganda and western studies, about 20% in the west compared to about 10% in Uganda, this suggests a generally lower rate of suicidal ideation in Uganda.

### 4.2. Prevalence of Suicidal Attempt in HIV/AIDS

Although the overall the rate of suicidal ideation and attempts was 10% (54/543), suicide attempts in the preceding year were reported as only 3.1%. This rate is very similar to the life time attempted suicide rate of 3.9% that was reported by [30] in semiurban Entebbe, Uganda. Other studies mainly from the west have reported higher rates such as O'Dowd et al. [36] in the United States who reported a rate of attempted suicide of 41.5%. Apart from the fact that the study participants in O'Dowd et al.'s [36] study were self-referred for psychiatric problems, they were mainly derived from subpopulations who are already at high risk for psychopathology [36].

The explanation for the difference between the rates of attempted suicide in the west with those in the Sub-Saharan African setting of Uganda cannot be explained alone by the differences in psychopathology of the base populations. Schlebusch and Vawda [13] who did their study among a predominantly heterosexual population in Kwazulu-Natal, South Africa, reported a rate of attempted suicide of 18.9% among 112 general hospital HIV in-patients [13]. These rates are similar to those seen in the west. These results from rural South Africa indicate that the prevalence of attempted suicide and possibly suicidal ideation in HIV/AIDS are determined by the complex interactions of factors, including the base psychopathology of the parent population.

### 4.3. Sociodemographic Correlates of Suicidal Ideation and Attempts

The sociodemographic characteristics found in the study were representative of the general population in south western Uganda. In this study there was a slight preponderance of females to males just as in the general population of Uganda, where females account for 51% and males 49%. Although slightly more females (10.7%) than males (7.6%) were suicidal, the difference was not statistically significant. Sociodemographic factors were not associated with suicidal ideation and attempts. Previous studies among HIV-positive persons found the following sociodemographic factors to be significantly associated with suicidal ideation and attempts: female gender [37–39]; male gender [38, 40, 41]; age [41, 42]; marital status [39]; and occupation [43].

Studies elsewhere have also reported the following sociodemographic factors to be associated with suicidal ideation and attempt in HIV/AIDS: lack of religious affiliation [44]; low levels of education [45, 46]; high levels of education [47]; unemployment [48–50]; widowhood [51]; and among the divorced [52]. Ecological factors represented by district in this study were not significantly associated with suicidal ideation and attempts.

### 4.4. Psychological Factors Associated with Suicidal Ideation and Attempt in HIV/AIDS

In this study the following psychological factors were significantly associated with suicidal ideation and attempts: depression, anxiety disorder, hopelessness, state/trait anger, and feeling style and sensing style of solving problems. Similar to this study, a previous study among HIV-positive persons in Uganda reported the following psychological factors to be associated with suicidal ideation and attempts: negative coping style, the psychiatric diagnoses of PTSD, generalized anxiety disorder, and major depressive disorder [30]. Other studies elsewhere have reported the following psychological factors to be associated with suicidal ideation and attempts: major depressive disorder, drug dependence and depressive personality, and dependent personality. The findings from this and previous studies emphasize the importance of negative psychological factors in the development of suicidal ideation and attempt in HIV/AIDS.

Participants who predominantly used the sensing style to perceive information and issues or who used the feeling style to make decisions and conclusions were more likely to be suicidal than those who used the intuitive style or made decisions using the thinking style. Kinyanda and colleagues [30], in semiurban Entebbe, reported that participants with higher negative coping style scores had higher rates of suicidal ideation and attempts than those with lower rates. In the west, Kalichman et al. [53] and Kelly et al. [33] reported that escape avoidance as a means of problem solving was associated with increased risk of suicidal ideation and attempts [33, 53].

### 4.5. Social Factors Associated with Suicide Ideation and Attempt in HIV/AIDS

In this study the following social factors were significantly associated with suicidal ideation and attempts: lack of social support, HIV stigma, being assaulted due to HIV and negative life events associated with children, and low perceived need for moral and practical social support. Previous studies among HIV-positive persons reported the following social factors to be associated with suicidal ideation and attempts: food insecurity; increasing number of negative life events and increasing stress scores [30]; physical and sexual abuse [43]; socioeconomic pressures and relational problems [13]; HIV stigma [54–59]; negative life events [30, 60–62]. Kinyanda and colleagues [61] among a general hospital population which was not assessed for HIV status in urban Kampala reported the following additional social factors to be associated with attempted suicide: living in overcrowded tenements (*Mizigos*), negative life events in childhood, negative life events in later in life, and negative life events in the previous year [61]. Social factors were found to be significant contributors to suicidal ideation and attempt in this study just like in many other studies of HIV populations. Although social factors were associated with suicidal ideation and attempt individually, in combination with other factors these relationships did not remain significant.

## 5. Conclusions

There is a considerable burden of suicidal ideation and attempts among individuals living with HIV/AIDS in south western Uganda. While the prevalence of suicidal ideation and suicide attempts in this study is similar to what has been reported in another study in Uganda, it is much lower than rates reported in western settings. This may reflect differences in the rate of psychopathology in the base populations.

## Figures and Tables

**Table 1:** Demographic characteristics of participants (*N* = 543).

	Frequency (*n*)	Percent (%)
District		
Mbarara	332	61.1
Isingiro	104	19.2
Bushenyi	45	8.3
Ntungamo	20	3.7
Other	42	7.7
Study site		
ISS clinic	304	56.0
TASO clinic	239	44.0
Sex of participant		
Male	131	24.1
Female	412	75.9
Tribe		
Munyankore	423	77.9
Mukiga	51	9.4
Muganda	33	6.1
Other	36	6.6
Religion		
Anglican	273	50.3
Catholic	175	32.2
Saved	45	8.3
Moslem	45	8.3
Other	5	0.9
Marital status		
Never married	63	11.6
Married/cohabiting	223	41.1
Widowed, separated, or divorced	257	47.3
Level of education		
No formal education	107	19.7
Primary	284	52.3
O' level	107	19.7
A' level	13	2.4
Vocational education	8	1.5
Tertiary/university	24	4.4
Employment status		
Formal employment or full-time business	169	31.1
Peasant farmer	259	47.7
Unemployed, homemaker, or retired	46	8.5
Student and any other	69	12.7

**Table 2:** Psychological correlates of suicidal ideation and attempt in HIV infected patients in HIV clinics in Mbarara (*N* = 226).

Independent variable	Suicidal (*n* = 25)- mean (SD)	Nonsuicidal (*n* = 201)- mean (SD)	cOR (95% CI)	*p* value	aOR (95% CI)	*p* value
Emotional factors						
Anxiety (BAI) score	18.64 (17.70)	7.98 (9.87)	1.06 (1.03–1.09)	0.001^*∗*^	1.00 (0.95–1.06)	0.898
Depression (BDI) score	10.24 (7.89)	4.47 (5.60)	1.13 (1.07–1.20)	0.001^*∗*^	1.06 (0.97–1.17)	0.219
Hopelessness (BHS) score	4.52 (5.38)	2.79 (3.14)	1.12 (1.02–1.23)	0.024^*∗*^	1.03 (0.90–1.17)	0.682
Trait anger score	20.32 (6.81)	15.96 (5.90)	1.11 (1.04–1.16)	0.002^*∗*^	1.03 (0.96–1.13)	0.565
State anger score	13.92 (7.46)	10.45 (1.78)	1.24 (1.16–1.38)	0.001^*∗*^	1.17 (1.01–1.34)	0.032^*∗*^
Problem solving styles						
Sensing style score	19.00 (2.71)	20.62 (3.33)	0.87 (0.77–0.98)	0.024^*∗*^	0.92 (0.77–1.13)	0.465
Intuitive style score	19.60 (3.16)	20.84 (3.20)	0.89 (0.79–1.01)	0.073		
Feeling style score	19.68 (3.34)	21.16 (3.15)	0.88 (0.78–0.99)	0.034^*∗*^	1.01 (0.84–1.23)	0.910
Thinking style score	20.92 (3.11)	21.32 (3.16)	0.96 (0.85–1.09)	0.545		

cOR: crude odds ratios; aOR: adjusted odds ratios.

^*∗*^ *p* < 0.05.

**Table 3:** Relationship between ways of coping and suicidal ideation and attempts among HIV infected patients in HIV clinics in Mbarara, July–October 2009 (*N* = 317).

Variable	Mean score (SD), suicidal	Mean score (SD) nonsuicidal	cOR (95% CI)	*p* value	aOR (95% CI)	*p* value
Seeking social support	7.14 (4.84)	9.66 (4.60)	0.89 (0.83–0.97)	0.006^*∗*^	0.92 (0.82–1.04)	0.189
Accepting responsibility	2.35 (2.55)	3.29 (2.90)	0.88 (0.76–1.03)	0.097		
Escape-avoidance	4.17 (4.42)	6.53 (5.41)	0.91 (0.84–0.99)	0.027^*∗*^	0.98 (0.88–1.26)	0.588
Planful problem solving	4.76 (3.82)	6.33 (3.88)	0.89 (0.79–0.99)	0.036^*∗*^	1.05 (0.83–1.15)	0.780
Positive reappraisal	5.57 (4.19)	8.24 (5.11)	0.90 (0.83–0.97)	0.009^*∗*^	0.96 (0.80–1.16)	0.657
Confrontive coping	3.59 (3.96)	5.02 (4.38)	0.92 (0.84–1.01)	0.093		
Distancing	3.21 (3.28)	5.04 (4.30)	0.89 (0.80–0.99)	0.026^*∗*^	0.96 (0.76–1.23)	0.761
Self-controlling	3.93 (3.81)	5.35 (4.61)	0.93 (0.844–1.02)	0.110		

^*∗*^ *p* < 0.05.

**Table 4:** Association between suicidal ideation and attempt and aspects of social support in HIV infected patients in HIV clinics in Mbarara, July–October 2009 (*N* = 317).

Variable	Suicidal *n* (%) (*n* = 29)	cOR (95% CI)	*p* value	aOR (95% CI)	*p* value
Needs support					
Yes	24 (8.1)	0.30 (0.10–0.89)	0.030^*∗*^	1.39 (0.23–8.57)	0.721
No	5 (22.7)	1			
Receives support					
Yes	23 (8.3)	0.50 (0.19–1.31)	0.156		
No	6 (15.4)	1			
Needed by others					
Yes	21 (7.2)	0.19 (0.07–0.47)	0.000^*∗*^	0.32 (0.05–2.19)	0.246
No	8 (29.6)	1			
Gives needed support					
Yes	19 (7.1)	0.31 (0.13–0.71)	0.006^*∗*^	0.66 (0.15–2.87)	0.583
No	10 (20.0)	1			
Isolating self					
Yes	7 (25.0)	0.25 (0.10–0.65)	0.004^*∗*^	0.49 (0.12–1.73)	0.245
No	22 (7.6)	1			
Ashamed of HIV					
Yes	11 (16.2)	2.48 (1.11–5.54)	0.027^*∗*^	1.44 (0.51–4.08)	0.497
No	18 (7.2)	1			
Guilt of HIV positivity					
Yes	9 (12.7)	1.64 (0.71–3.78)	0.246		
No	20 (8.1)	1			
Blaming self for HIV					
Yes	9 (11.7)	1.46 (0.63–3.35)	0.374		
No	20 (8.3)				
Physically assaulted					
Yes	3 (33.3)	5.42 (1.28–22.96)	0.022^*∗*^	6.66 (1.48–30.08)	0.014^*∗*^
No	26 (8.4)				

^*∗*^ *p* < 0.05.

## Additional Points

This study was facility based and the findings may not be generalizable to the general population. In addition, the study was cross-sectional in nature. Therefore, causality could not be determined.
